# Supplementary material for: Causal Relationship between Aging and Anorexia Nervosa: A White-Matter-Microstructure-Mediated Mendelian Randomization Analysis
Source: Biomedicines. 2024 Aug 16;12(8):1874. doi: 10.3390/biomedicines12081874 (PMC11351342; doi:10.3390/biomedicines12081874)
Supplement: Supplementary file 1 [file biomedicines-12-01874-s001.zip › Supplementary Figure/Figure S9/Leaveoneout plot of MR analysis for ubm-b-1879 to AN.pdf]

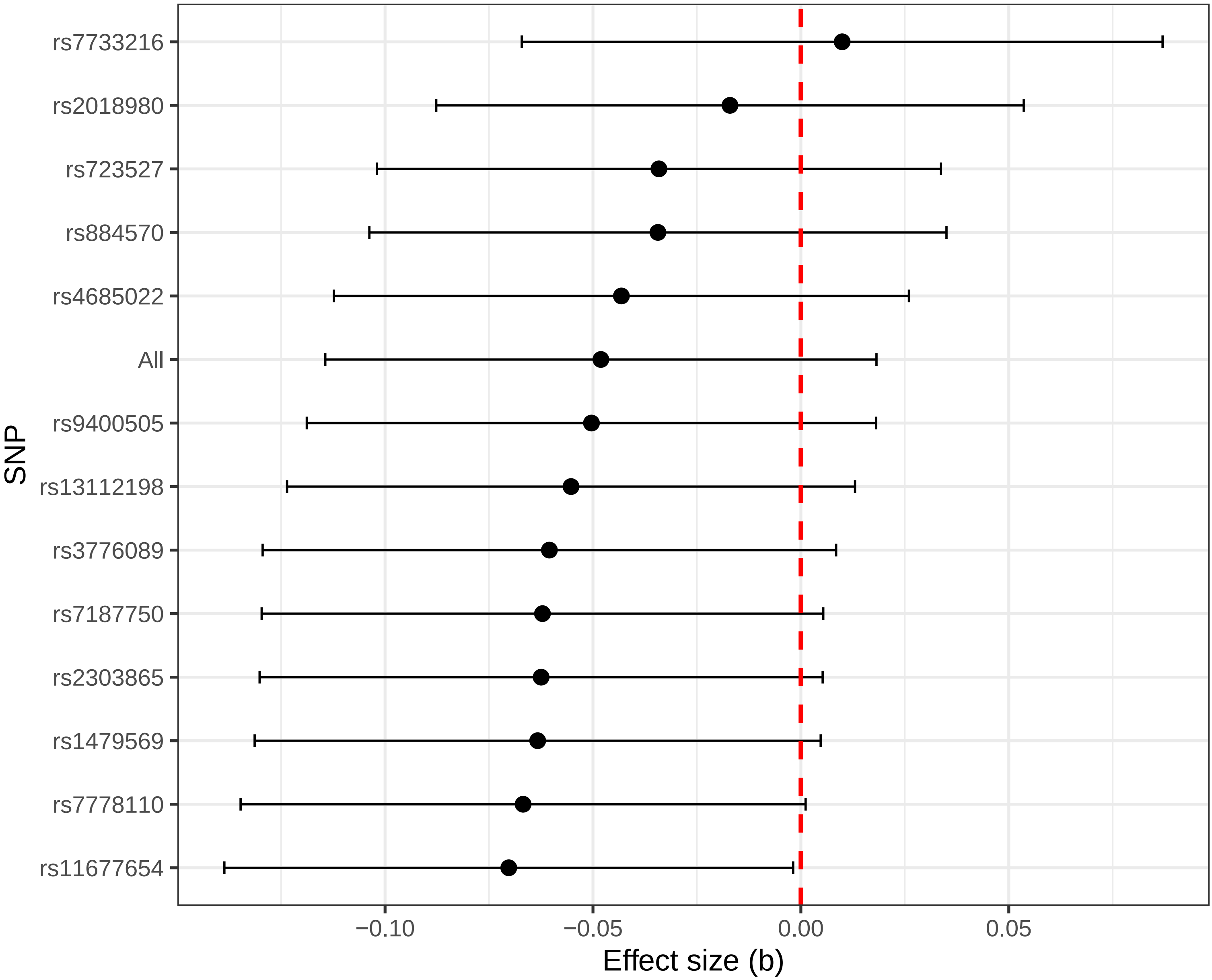
rs7733216
rs2018980
rs723527
rs884570
rs4685022
All
rs9400505
rs13112198
rs3776089
rs7187750
rs2303865
rs1479569
rs7778110
rs11677654
SNP
−0.10
−0.05
0.00
0.05
Effect size (b)
